# Sex Associated Effects of Noise Pollution in Stone Sculpin (*Paracottus knerii*) as a Model Object in the Context of Human-Induced Rapid Environmental Change

**DOI:** 10.3390/biology10101063

**Published:** 2021-10-19

**Authors:** Yulia P. Sapozhnikova, Anastasia G. Koroleva, Vera M. Yakhnenko, Igor V. Khanaev, Olga Yu. Glyzina, Tatyana N. Avezova, Aleksandra A. Volkova, Angela V. Mushinskaya, Marina L. Tyagun, Artem N. Shagun, Mikhail M. Makarov, Sergey V. Kirilchik, Nikolay P. Sudakov, Igor V. Klimenkov, Lyubov V. Sukhanova

**Affiliations:** 1 Limnological Institute SB RAS, 3 Ulan-Batorskaya, 664033 Irkutsk, Russia; ankor-2015@yandex.ru (A.G.K.); vera@lin.irk.ru (V.M.Y.); igkhan@lin.irk.ru (I.V.K.); glyzina@lin.irk.ru (O.Y.G.); fototanya@mail.ru (T.N.A.); alexa.volkova8@lin.irk.ru (A.A.V.); m.angel.v@mail.ru (A.V.M.); mary@lin.irk.ru (M.L.T.); mmmsoft@hlserver.lin.irk.ru (M.M.M.); kir@lin.irk.ru (S.V.K.); npsudakov@gmail.com (N.P.S.); iklimen@lin.irk.ru (I.V.K.); lsukhanova@yandex.ru (L.V.S.); 2 Institute of the Earth’s Crust SB RAS, 128 Lermontova Street, 664033 Irkutsk, Russia; shagun@crust.irk.ru

**Keywords:** noise pollution, HIREC, blood parameters, hair cell loss, telomeres, stress-induced senescence

## Abstract

**Simple Summary:**

In this comprehensive multidisciplinary study, we applied a novel multilevel approach to stone sculpins *Paracottus knerii* Dybowski, 1874, as model organisms and test for the first time the hypothesis of sex-dependent differences in response to long-term noise exposure in fish. The results testify that the stone sculpin females appeared to experience excessive stress, while the males showed adaptive recalibrations. These effects may be explained by a unique adaptive strategy of offspring care in the stone sculpin males and their biological role in reproductive behavior within the species. The findings obtained may help to elucidate the links between noise exposure in the context of human-induced rapid environmental change (HIREC), long-term sex-related changes in fishes, and the possible further evolutionary success of a species. Such HIREC modeling not only provides information about the potential consequences under anthropogenic pressure but also can help identify the natural mechanisms of stress resistance in different species, including those related to sex, and also contribute to the development of effective environmental management practices.

**Abstract:**

This work simulates the consequences of HIREC using stone sculpins as model organisms. Sex-dependent effects of long-term noise exposure at mean sound pressure levels of 160–179 dB re 1 μPa (SPL_pk–pk_) were measured. We applied a multilevel approach to testing the stress response: a comparative analysis of the macula sacculi and an assessment of hematological and molecular stress responses. Noise exposure resulted in hair cell loss, changes in some cytometric parameters in blood, and an increase in the number of functionally active mitochondria in the red blood cells of males and its decrease in females, demonstrating a mitochondrial allostatic load and depletion of functional reserve. Finally, a statistically significant decrease in the telomerase activity of the auditory epithelium and a shortening of telomere length in the brain as molecular markers of stress were observed after noise exposure only in females. No significant decrease in telomerase activity and shortening of telomere length in nerve target tissues were observed in stressed males. However, we recorded an increase in the telomerase activity in male gonads. This sex-dependent difference in load may be associated with accelerated cellular aging in females and lower stress-related long-term risk in males. In this article, we discuss possible reasons for these noise-induced stress effects.

## 1. Introduction

The problem of noise in the underwater environment was discussed at the 19th meeting of the United Nations Open-ended Informal Consultative Process on Oceans and the Law of the Sea (ICP-19) where the negative consequences of the impact of anthropogenic noise on aquatic life were noted, including disruption of communication between individuals, displacement of animals from their breeding and feeding grounds, and the occurrence of stress and disease leading to death [1]. Ship noise is considered the most widespread continuous underwater pollutant, and it can completely dominate low-frequency (up to 500 Hz) soundscapes in heavily trafficked areas [2,3]. The passage of a ship is described by a broadband noise with dominant tonal (narrowband) components [4,5]. Moreover, extensive data analysis reveals that half of the total power radiated by a modern fleet comes from 15% of the vessels, mainly those with source levels above 179 dB re 1 μPa at 1 m [1,6]. However, underwater ambient noise levels have doubled every decade since 1963 in the low-frequency bands attributed to shipping [6,7], possibly leading to the emergence of a stress phenotype in different species due to human-induced rapid environmental change (HIREC) [8,9]. While the consequences of the other stressors, such as carbon dioxide or persistent organic pollutants, are usually obvious, and the pollutants persist in the environment for some time, the long-term consequences of noise on aquatic life are not yet clear [7,9,10,11].

Studies of acoustic effects on aquatic organisms, especially fish, show that anthropogenic noise negatively influences both teleost and elasmobranch fish [7,12,13] and directly affects vocal teleost fish, increasing stress, altering their metabolic processes, and negatively affecting all stages of their life activity [5,14,15]. Most fish communicate and hear best precisely in the range of technogenic noise, below 500 Hz, by sensing particle accelerations with their inner ear [16,17]. However, very little is known about stress effects in different fish species, and the consequences of such impacts in response to human-induced noise are even less clear [10,18].

The higher-stress situations may have maladaptive or negative consequences (distress), including pathogenic effects [19]. It is also worth noting that the consequences of prolonged stress are particularly deleterious due to energetic costs associated with coping with the stress response (allostatic load), negatively affecting other vital functions, including reproduction [20] and enabling changes at the population and species levels [9,21]. In this regard, the response to stress plays a critical role in an organism’s ability to survive the challenge of homeostasis [22,23].

The stress response is initiated and controlled by hormonal systems that regulate secondary and long-term tertiary stress response factors [24]. However, the high cortisol levels in teleost fish do not last for a long time and may return to initial levels as soon as the animal adapts to the stressors [25]. Interestingly, in the context of stress regulation, all steroid hormones, including glucocorticoids and sex hormones, synthesize in a process that is regulated by mitochondria [26,27], further linking mitochondrial biology to stress signaling. Thus, mitochondria have recently been considered a key element of the stress response (1) as a stress target and (2) as a mediator of stress pathophysiology and, hence, a precursor of stress-induced molecular and cellular changes [28,29].

Nonetheless, recent studies of aquatic animals, particularly fish, have been primarily focused on initial inflammatory and oxidative stress pathways as a result of noise exposure [30,31,32], while data linking noise exposure to measures such as mitochondrial alterations, DNA methylation, and telomere length are almost absent. There are few studies indicating that noise exposure, similar to air pollution, may affect the health of humans, birds, or mollusks via DNA methylation and telomere length [19,33,34,35,36]. Notably, these studies have mostly used telomere length as a biomarker for the senescence process without investigating the links between biological markers of senescence and hearing loss.

The exposure time of a stressor can also be important: vertebrates from numerous taxonomic groups have been proven to be stress-resilient during the breeding season [5,23,37]. Moreover, most species appear to be even less sensitive to high stress levels in the post-spawning period, during parental care; even the species that have a restricted spawning season and are tied to specific spawning grounds and cannot relocate [5]. Nevertheless, there are few studies of noise effects on fish during the period of parental care. In birds, particularly, in western sandpipers, *Calidris mauri* [38], and American tree sparrows, *Spizella arborea* [39], the sex-performing parental care is more stress-resistant, suggesting that parental care reduces physiological responses to stress. However, harsh stress may lead to the reactivation of stress sensitivity, which may result in the abandonment of current broods for future reproduction [40]. Brood abandonment is expected only when the cost-benefit ratio for current broods is high compared to the potential for future reproduction [41]. In this context, stress induced by noise is expected to have a lower effect during the period of parental care, unless it is extreme enough to distract fish from courtship and exceeds a certain threshold [5].

According to the concept of the stressotope, variations within the stress response are also introduced by the environmental history of the fish [9]. Therefore, critical changes that occurred previously could lead individuals of the species into the dependence curve, making the stress picture ambiguous. Thus, to rule out this multi-stress effect and establish causality in responses to treatment, the question of adequate control in the experiment arises [42]. In Lake Baikal, the World Heritage Center, shipping traffic is considered not very active, there are no seismic prospecting activities, and natural sounds dominate [43,44,45]. This allows us to consider Lake Baikal a natural laboratory where acoustic experiments can be simulated to ensure pure and adequate control. In this case, we exclude additional anthropogenic stressors that may have occurred earlier, cannot be controlled, and involve a multi-stress effect. Because no evidence of differences between laboratory and field studies regarding the likelihood of an effect of noise on stress responses was previously found [5], we modeled under laboratory conditions specific generalized noise factors that are as close as possible to the anthropogenic noise in natural environments. The effects in the control and experimental groups are considered two independent scenarios: (1) without anthropogenic impact and (2) with HIREC.

Because of species-specific features in hearing systems, predicting what sound affects various species remains a complicated problem [7]. However, ship noise may cause strong behavioral effects even in fish with poor hearing [7,46,47]. Consequently, fish that use low-frequency signals, such as sculpins (Cottidae) [16,48,49,50], can be more affected by continuous human-induced noise than fish that use high-frequency signals [5,51].

In this regard, sculpins represent the most appropriate model to study the potential resistance or susceptibility to stress in fish, as induced by prolonged noise. First, sculpins are widely distributed worldwide and are actively used as model fish in various experiments [14,49,50]. Second, sculpins are capable of vocalization and hear low frequencies in the range coinciding with human-induced noise [14,49,50], as well as likely detect substrate vibrations carried out during sound production [48,52]. Additionally, these species have a restricted spawning period, and, thus, they can be trapped into enclosed spaces in their habitat by anthropogenic noise [7], especially during the paternal care period [5].

In Lake Baikal and some other surrounding water bodies, the stone sculpin (*Paracottus knerii* Dybowski, 1874) inhabiting depths of 40–50 m is the most abundant among the sculpins [53]. This species is characterized by significant differences in the reproductive behavior of males and females. The post-spawning females immediately retreat to great depths, and the males guard the clutches until the juveniles hatch, as they do not have an opportunity to relocate during the paternal care period [53,54]. The protection of eggs by males is undoubtedly very effective. The stone sculpin males protect the eggs from predators and apparently aerate them with the movement of their pectoral fins. No mass mortality of males or females is observed after spawning [54], probably indicating sufficient stress resistance of the species.

In this novel multilevel study, we test the hypothesis of sex differences in response and stress-resistance to long-term noise exposure using the Baikal stone sculpin species as a model organism. A continuous broadband noise with the dominant tonal (narrowband) components from 10 to 500 Hz was selected according to the adequacy of this stimulus in modeling anthropogenic noise pollution [1,5,10]. The duration of the experiment (up to seven days) is long-term [55] and appropriate for the study of the chronic stress responses [56,57,58,59] considering cumulative and synergistic effects. Source levels can range from 150 dB to over 190 dB re 1 μPa at 1 m for the largest vessels [4] and have recently averaged 179 dB [1,6]. Moreover, such levels are considered a possible threshold for acoustic stress in fish, although the authors note that more data are needed to confirm this suggestion [7,10,57]. Thus, in the present work, the intensity level (160–179 dB re 1 μPa SPL_pk–pk_) is considered a threshold because we study the effects of excessive noise on fish. A detailed, comprehensive analysis of sensory auditory epithelium and blood parameters and an assessment of potential molecular stress were carried out for this species. As a secondary hematological stress indicator, we examined blood cells, particularly the allostatic load on mitochondria, multifunctional life- and energy-sustaining organelles [60,61]. Molecular stress was estimated using methods to measure telomerase activity and telomere length, which serve as markers of the physiological state of an organism [62,63].

## 2. Materials and Methods

### 2.1. Experimental Animals

Spawning of the stone sculpins takes place in a limited period from May to July at shores to a depth of 1.5 m on specific rocky or stony–sandy ground [53,54]. The age of spawning individuals is 2+ to 4+ years, with three-year-old fish predominating and accounting for over 60% of all fish caught. The number of males in relation to females in the natural environment is usually large, with a ratio of 2:1 [54]. In the second half of summer and in autumn (post-spawning period), this species is found at depths of more than 10 m [54].

Overall, 36 adult mature stone sculpins sampled in late summer 2020 from the coastal zone of Lake Baikal in the area of the Limnological Institute’s station at the Bolshiye Koty settlement were used for this study (number of specimens: 18 (♀) and 18 (♂); average length, TL, cm: 81.47 ± 8.93 (♀) and 82.16 ± 14.28 (♂); weight, g: 8.09 ± 3.09 (♀) and 9.27 ± 4.56 (♂); age by otoliths, years: 3+–4+). The sex ratio both in the control and experimental tanks was about 2:1 (males:females) because we used randomly sampled fish from the natural environment. Nevertheless, for the correct statistical calculation, later, we sampled males and females in the ratio of 1:1. We should clarify that the lack of replicates is a weakness of the study.

**Figure 1:**
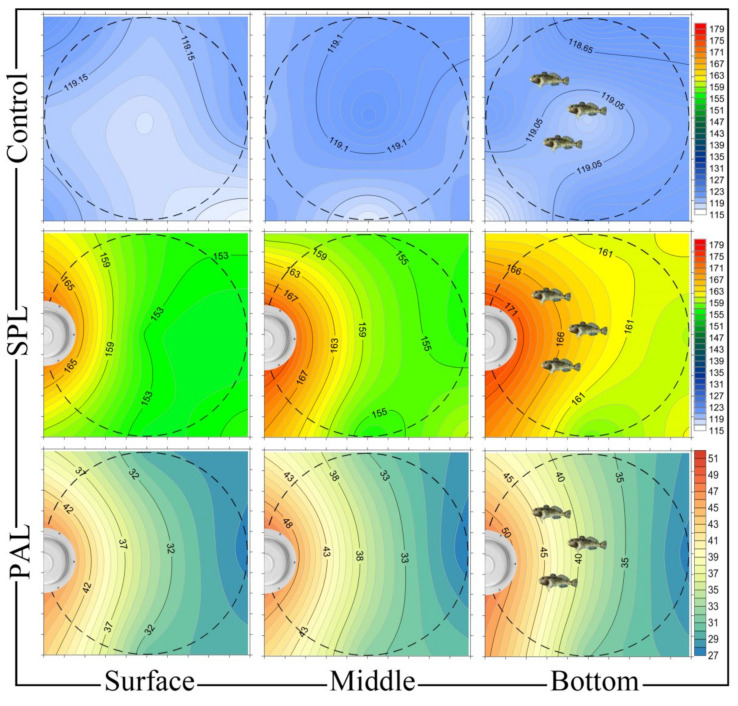
Sound pressure levels (SPL, dB re: 1 μPa) and the particle acceleration levels (PAL, dB re: 1 μm/s^2^) in the *x*, *y*, and *z* directions at the surface, mid-depth, and tank bottom. The control tank was used for fish without anthropogenic impact, and the experimental one for fish with noise exposure (HIREC effect).

All stone sculpins were identically acclimated prior to the start of the experiment. In particular, they were kept at 12 °C and a 12:12 h light/dark cycle, with a daily water change of 30%; the same conditions were strictly maintained during the experiment. Fish were kept under controlled conditions at the Experimental Freshwater Aquarium Complex for Baikal Hydrobionts at Limnological Institute (LIN SB RAS). To conduct the experiments on the effects of noise-induced stress in stone sculpins, two circular polypropylene tanks were set up in separate rooms (1000 L of water, sufficient space to avoid direct excessive exposure). The first tank was used for the control fish (N = 18, without noise exposure) and the second for the experimental fish (N = 18, with noise exposure). Fish were mostly localized on the small stones (identical to the natural substrate), which were placed at the bottom midpoint of the tanks (Figure 1).

### 2.2. Noise Exposure

We established a protocol for technogenic (anthropogenic) noise exposure consisting of 2 min noise events interspersed with the rest periods corresponding to the natural soundscape of this species in fast-flowing river waters and the coastal zone of freshwater bodies [64,65,66]. We aimed to determine the effects of noise exposure on stress (maximum sound pressure level (SPL_pk-pk_) of 179 dB re 1 μPa, mean 167 dB re 1 μPa, 115–120 dB re 1 μPa in the rest periods for 2 s and in the control tank). The noise events were repeated continuously for seven days. Experimental and control stone sculpins were sampled on the seventh day after the onset of noise exposure.

A broadband noise with the dominant tonal (narrowband) components from 10 to 500 Hz was generated by a USUS gaming laptop (ROS, the STRIX RAID DLX sound card) using an amplifier (VOLTA PA100). The UW30 Electro-Voice hydroacoustic underwater speaker was placed under the water at half the depth of the experimental tank.

The RESON TC4013 piezoceramic hydrophone was used to verify the acoustic signal. To amplify the signal from the hydrophone, a linear amplifier was developed in the Hydrology and Hydrophysics Laboratory of LIN SB RAS and calibrated using a GSS-10 arbitrary waveform generator (Russia) and a Tektronix MSO2012 digital oscilloscope (USA). Then, the acoustic signal was fed into an LCard E-440D analog-to-digital converter and analyzed using the LCard PowerGraph software. The radiation of sound intensity, its uniformity, and particle motion were measured at different points of the tanks, both in the vertical plane (surface, center, and bottom) and in the horizontal plane (nine squares) (Figure 1). The average and maximum intensity levels were 167 and 179 dB re 1 μPa, respectively, at the tank bottom, where most of the experimental fish were located.

Particle acceleration was measured using a “Baikal7-HR” triaxial digital recorder with piezoelectric A1638 accelerometers (Moscow, Zelenograd, Russia), as described previously [59]. The amplifiers of the “Baikal-7HR” and the recording elements of the A1638 accelerometers were calibrated with a precision signal generator (G3-110), a source of sinusoidal electrical oscillations with high accuracy and frequency stability in the range of 0.01 Hz–2 MHz. During calibration, the correction coefficients of each orthogonally independent component (*x*, *y*, and *z*) were calculated to correctly determine the amplitude-frequency spectrum of the acceleration. To collect the particle acceleration data, the accelerometer was placed at the same positions of the tank as the hydrophone, both in the vertical plane (surface, center, and bottom) and in the horizontal plane (nine squares). The particle acceleration measurements (μm/s^2^) were recorded for each of the three accelerometer axes (*x*, *y*, and *z*) and recalculated to particle acceleration level (PAL, dB re: 1 μm/s^2^) according to Equation (1) [67]. Figure 1 shows the sound pressure levels (SPL, dB re: 1 μPa) and the particle acceleration levels (PAL, dB re: 1 μm/s^2^) in the *x*, *y*, and *z* directions at the surface, mid-depth, and tank bottom.

(1) $$\mathrm{PAL}=20\log_{10}\left(\sqrt{x^{2}+y^{2}+z^{2}}\right)$$

### 2.3. Ethical Standards and Tissue Collection

The experiments and sampling were conducted in accordance with the animal welfare laws, guidelines, and policies of Russia and approved by the Ethics Committee of Limnological Institute SB RAS. Fish from the control and noise-exposed tanks were collected with a dip net on the seventh day after the start of the experiment. Stone sculpins from both tanks were anesthetized with clove oil (0.02–0.05 mL/L, a sedative containing eugenol) and then euthanized with tricaine mesylate (MS222) according to the AVMA Guidelines for the Euthanasia of Animals, 2020 and [68]. Blood samples, inner ear tissue (macula sacculi), the brain (medulla oblongata), dorsal musculature, gonads, and dorsal fins were collected from the euthanized fish. The crania of all fishes were dissected from the ventral side, and the brains and right and left labyrinths were removed. The macula sacculi were extracted along with the sagittal otoliths, which were used to determine the age of each fish.

### 2.4. Blood Parameters 

Smears of peripheral blood and hematopoietic organs (kidneys and spleen) in both males and females were stained with azure eosin and examined with an Axiostar plus light microscope equipped with an AxioCam ICc1 camera (Zeiss, Jena, Germany) to identify the image of inflammatory processes and expressions of oxidative stress. Cytometric parameters were measured in peripheral blood (erythroblasts, mature red blood cells, lymphocytes, monocytes, and neutrophils) and in the spleen and kidney (blasts, phagocytes, lymphocytes, and plasmocytes). Cell size was evaluated using the Image-Pro Plus software. Additionally, cell area (S), nuclear area (s), and nuclear-to-cytoplasmic ratio (NCR) were calculated for red blood cells using the formula for the area of an ellipse. Numerical eccentricity (E) was used as a quantitative characteristic of red blood cell shape [59,69]. Red blood cell hemoglobin protein (Hb) was extracted and analyzed using previously tested methods [59].

We estimated the mitochondrial membrane potential of red blood cells as a marker of red blood cell condition using methods that had been previously tested in other species [59,70]. In brief, red blood cells were incubated in Medium 199 containing Hank’s salts (PanEko, Moscow, Russia, Cat. No. S230p) and MitoTracker Orange (100–500 nM, Life Technologies, Waltham, MA, USA) at 37 °C for 25 min, then fixed with 2% paraformaldehyde for 15 min. The nuclei were stained with DAPI solution (10 μg/mL, in PBS) (Sigma-Aldrich, Burlington, MA, USA, Cat. No. D9542) for 15 min. The resulting preparations were coverslipped with the ProLong Gold antifade reagent (Life Technologies) and examined on an LSM 710 laser confocal microscope (Zeiss). Confocal images were processed using two programs: ZEN 2010 (Zeiss) and Imaris Bitplane 7.2.3. Red blood cells were analyzed layer by layer in the form of 2D slices, Z-stacks volume. Using the Imaris Bitplane 7.2.3 software package, we separated Z-stacks into smaller fragments and determined the volumes occupied by the MitoTracker Orange fluorescent marker. In the selected area, the entire array of fluorescent signals was highlighted, the volume of which was then automatically summed. The total volume of mitochondria was recalculated into the relative one for all fragments and converted to represent a convenient 1 × 10^6^ μm^3^ (100 μm × 100 μm) volume.

### 2.5. Inner Ear Analysis

The macula sacculi were fixed according to previously tested methods and analyzed by confocal microscopy [59,71]. The sensory epithelium of the inner ear of each fish was incubated for 30 min in 2% paraformaldehyde (Sigma-Aldrich, Burlington, MA, USA, Cat. No. 158127) in 0.1 M phosphate buffer (PBS) (pH 7.4) and permeabilized for 20 min in 0.25% Triton X-100 (Sigma-Aldrich, Burlington, MA, USA, Cat. No. T8787). Actin microfilaments were stained for 40 min with Alexa Fluor™ 488 Phalloidin (Sigma-Aldrich, Burlington, MA, USA, Cat. No. P5282) with methanol (40× stock solution) diluted in phosphate buffer (5 µL/200 µL). Then, the samples were washed three times in Hank’s solution (PanEko, Moscow, Russia, Cat. No P020p). The stained samples were mounted on glass slides in ProLong Gold Antifade Mountant (Thermo Fisher Scientific Inc., Waltham, MA, USA, Cat. No. P36930), covered with a coverslip, and analyzed by confocal microscopy (LSM 710, Carl Zeiss).

Hair cell density on the macula sacculi was determined from five preselected areas at 5%, 25%, 50%, 75%, and 95% of the distance along the rostral-caudal axis of the macula sacculi, as in previous studies [59,71]. In maculae with some small cracks at the measurement area, the hair cell density was calculated with a slight displacement relative to the rostral-caudal axis. The number of hair cell bundles was quantified in each area in control and noise-exposed stone sculpins. Then, the 100 μm × 100 μm counting boxes were placed over digital images in ZEN 2010 (Zeiss). Hair cell density of intact hair bundles was counted in each area and converted to represent a convenient 2500 µm^2^ (50 μm × 50 μm) area.

### 2.6. Telomere Length and Telomerase Activity Analysis

Telomere length was quantified by polymerase chain reaction (qPCR). Genomic DNA from each sample of the inner ear, brain (medulla oblongata), white muscle (dorsal musculature just below the dorsal fin), and gonads of control and experimental stone sculpins was extracted using the phenol/chloroform method [72,73]. Relative telomere length (telomere DNA concentration/single-copy gene (DNA) concentration, T/S) was measured by qPCR as described in Cawthon [74] using Rotor-Gene Q 6000 (QIAGEN, Germany). The RAG1 gene served as the reference gene. Primer pairs were designed in Primer Blast using the gene sequence of the *Paracottus knerii* (LC125868) stone sculpin. The primer sequences were GGAGACCCAGACAACGATGG (forward) and CGGCTGGGTTTGACCTTTTG (reverse). The qPCR mix contained 1× Snp-buffer, 0.25 mM dNTPs, 0.2 U Snp-polymerase and 2.5 mM MgCl2 (Evrogen, Russia), 0.2–0.3 ng DNA, 0.5-fold SYBR Green (Lumiprobe, Hunt Valley, MD, USA), and 0.5 pmol of each RAG1 primer. For qPCR of telomere repeats, 0.17 pmol Tel1 and 0.5 pmol Tel2 primers were added to the reaction mixture instead of the RAG1 primers. DNA polymerase was activated at 95 °C for 3 min. The telomere reaction was immediately subjected to 45 cycles at 95 °C for 15 s and at 54 °C for 2 min. Touchdown PCR was used for amplification of the reference gene fragment by gradually lowering the primer-annealing temperature from 71 to 64 °C over the first seven cycles. For RAG1, one cycle included the following steps: 95 °C for 10 s, 64 °C for 15 s, and 72 °C for 15 s. The cycle was repeated 35 times. The measurement of a telomere length for each sample was repeated three times by qPCR. T/S values are presented as mean ± SD.

Then, we quantified telomerase activity using a real-time telomere repeat amplification protocol (Q-TRAP) assay [59]. Total protein from the inner ear, brain (medulla oblongata), white muscle (dorsal musculature just below the dorsal fin), and gonads was isolated using CHAPS buffer as described in Yip et al. [75]. We used acetonitrile (Cryochrom, St. Petersburg, Russia) to purify the protein mixture from the CHAPS buffer components for the more accurate concentration measurement; 200 µL of acetonitrile was added to 50 µL of the protein mixture and centrifuged at 13.4× *g* for 10 min. The sediment was dissolved in 8M urea (Sigma-Aldrich), and then the protein concentration was quantified by the Bradford method [76] using a commercial assay (Sileks, Moscow, Russia). The real-time Q-TRAP assay was performed using the Rotor-Gene Q 6000 instrument (QIAGEN, Hilden, Germany) as described in Yip et al. [75] with some modifications. A measurement of 15 µL of the resultant solution contained one-fold buffer (Evrogen, Moscow, Russia), one-fold Encyclo DNA polymerase, 0.25 mM dNTPs, 0.5-fold SYBR Green, 1 pmol TS primer [77], 0.5 pmol ACX primer [78], and 200 ng of the protein mixture. The reaction began with incubation at 8–10 °C (to match stone sculpin habitat) for 30 min for TS primer extension by telomerase, followed by incubation at 94 °C for 10 min to inactivate telomerase and activate Encyclo polymerase. Samples were immediately used for 35 PCR cycles with the following parameters: 94 °C for 30 s, 58 °C for 30 s, and 72 °C for 1 min. A lysate-free control (containing all components except protein) was used as a no-template control. Each sample was run in duplicate. Cycling thresholds were determined from the semi-logarithmic amplification plots (logarithmic increase in fluorescence signal versus cycle number). Relative telomerase activity was calculated by the ΔΔCt method [79] using the Rotor Gene version 2.3.1 software. The telomerase activity of the first control fish was set as 1, and the remaining samples were calculated with reference to the selected control [75].

### 2.7. Statistical Analysis

Preliminary testing was carried out for homogeneity of variances (Breakdown and 1-way ANOVA, Brown-Forsythy test) and the samples normality (Shapiro-Wilk test). All the samples had distributions with equal variance (*p* > 0.05); however, some of them were not distributed according to the law of normal distribution (for blood parameters, telomere length, and telomerase activity). Thus, for reliability, we used nonparametric statistics. The differences between control and experiment and between the sexes in the experimental group in all tissues for blood parameters, telomere length, and telomerase activity analysis were estimated using a Kruskal-Wallis test (Statistica 10 software package). Hair bundle density between the control and noise-exposed stone sculpins was analyzed using 2-Way ANOVA, followed by the Tukey HSD test (the R software for statistical calculations). Results were considered statistically significant at *p* < 0.05.

## 3. Results and Discussion

### 3.1. Noise-Induced and Sex-Dependent Changes in Stone Sculpin Blood

Noise exposure led to changes in stone sculpin blood profiles, which showed adequate inflammatory responses in both males and females, as well as current oxidative stress reactions in females (Table 1). In particular, we observed high ratios of neutrophils to lymphocytes (N:L ratio) in female blood samples, which reliably indicate a chronic stress response [80]. Previously, this reaction was well-studied in certain species of fish and repeatedly proposed as an alternative method for measuring glucocorticoids [80,81,82]. However, due to the huge evolutionary differences between the great numbers of teleost species, responses to stress are highly variable, and this requires further detailed studies.

**Figure 2:**
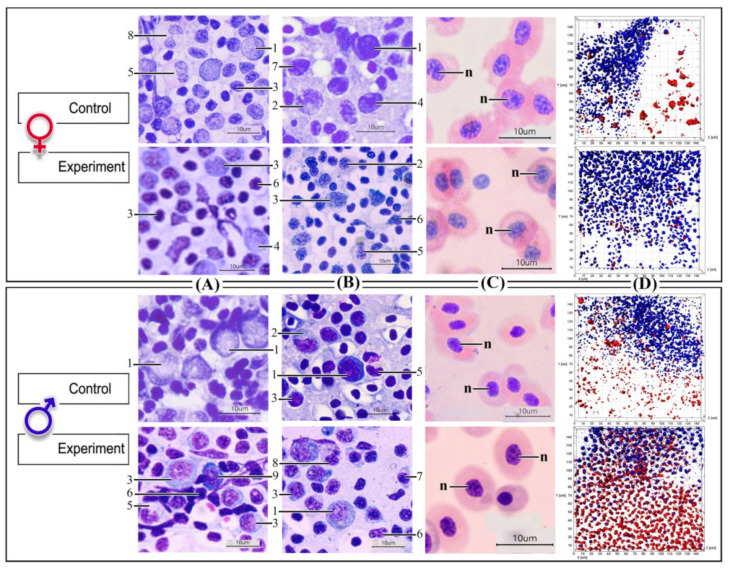
Blood parameters of stone sculpin males and females in control and seven days after the noise-exposure: (**A**,**B**) Cytometric parameters of blood cells in the hematopoietic organs (**A**—kidneys and **B**—spleen) of stone sculpin: 1—lymphoblasts, 2—myeloblasts, 3—prolymphocytes, 4—promyelocytes, 5—promonocytes, 6—lymphocytes, 7—myelocytes, 8—neutrophils, 9—plasmocytes (light microscopy); (**C**) Red blood cells in peripheral blood (light microscopy), n—red blood cell nucleus; (**D**) The volume of the fluorescent signal from native mitochondria (in red) of the red blood cells with a conserved membrane potential (functionally active mitochondria): staining for mitochondria (MitoTracker Orange CMTMRos, red) and for nuclei (DAPI, blue).

Stress-induced decreases in circulating lymphocyte numbers are due to glucocorticoid-induced changes in the reallocation of lymphocytes from the blood to various tissues [83]. Conversely, glucocorticoids activate an influx of neutrophils into the blood and reduce the efflux of neutrophils from the blood to other compartments [84]. Moreover, the change in the profile of mature blood cells in stone sculpin males and females compared to controls is in good agreement with an increase in the content of immature blasts of blood cells in the male kidneys and female spleen, the hematopoietic organs (Table 1, Figure 2A,B), as stress can increase erythropoiesis processes [85,86]. Such changes are thought to ensure that the various types of blood cells are directed to the tissues where they are needed during the stress response [87].

In addition to an increase in cell area and volume in females only, we also observed significantly elongated red blood cells (as a decrease in cell eccentricity) and larger nuclear areas and volumes in both noise-exposed male and female stone sculpins (Table 2, Figure 2C). Such changes may indicate recently increased metabolic activity in stressed stone sculpins, suggesting rapid systemic changes in response to noise exposure. Particularly, it has been previously suggested that larger nuclei may be associated with an increased ability to produce transcription products [59,88,89].

We then examined the volume of functionally active mitochondria in stone sculpin red blood cells. Examination of mitochondria with MitoTracker labeling showed that after long-term noise exposure, red blood cells of the stone sculpin males exhibited a 53–61% increase in the relative volume of active mitochondria compared to controls (*p* < 0.05; Table 2, Figure 2D). Previous studies also demonstrated significant adverse effects of stress on mitochondria and changes in function and size after stress [60,61], including sound-induced stress [59]. The rise in the relative volume of active mitochondria was referred to as an “enriched pool” of mitochondria with intact membrane potential [59,70]. Such an “enriched pool” of mitochondria in noise-exposed males could be associated with an increase in cellular energy cost at the initial phase of stress and subsequent adaptation. Previous observations suggest that several factors influence mitochondrial resistance. Thus, certain compounds, including antioxidants, conferred protection against stress-induced mitochondrial load and dysfunction [90,91], indicating the existence of stress-buffering factors in stone sculpin males.

On the contrary, the volume of functionally active mitochondria was reduced by 30–43% in the stone sculpin females compared to controls (*p* < 0.05; Table 2, Figure 2D). Such maladaptive mitochondrial changes are known as mitochondrial allostatic load and underlie functional recalibrations associated with stress pathophysiology [60]. A decrease in the volume of functionally active mitochondria in exposed stone sculpin females may be explained by altered energy cost with depletion of the organism’s resources during the exhaustion phase. These data are in good agreement with the fact that antioxidant enzymes may decrease under chronic restraint stress and other accompanying factors [92]. Previous human studies revealed that females with a severe stress load had white blood cells consuming more oxygen [93]. This finding may reflect an increased load on red blood cells and possible resulting mitochondrial dysfunction.

In addition to the changes in blood cytometric parameters, we also measured hemoglobin concentration and blood oxygen capacity and observed changes in hemoglobin fractions. The hemoglobin of the control stone sculpins contained only one cathodic fraction, whereas the noise-exposed fish had two cathodic fractions (Table 2). Additionally,, we recorded a significant increase in hemoglobin concentration and blood oxygen capacity in female blood (Table 2). These changes appear to be an adaptation of the oxygen transport system to the altered energy cost and increased load in tissues lacking oxygen under stress [94]. An increase in cathodic protein components may also improve fish survival in the stressotope. On the other hand, noise exposure can be considered a stressor that contributes to hemoglobin modification, consequently leading to a higher affinity for oxygen. Notably, mitochondria are the major producers of reactive oxygen species within the cell [95], which play signaling and other vital roles but can lead to oxidative stress when they suppress antioxidant defense mechanisms, playing a key role in neurodegenerative processes [96] and stress pathophysiology. Other animal models have also confirmed that changes in mitochondria directly influence telomere maintenance [97] and even the rate of aging and lifespan [98,99,100].

### 3.2. Noise Causes Saccular Hair Cell Loss in Both Male and Female Stone Sculpins

**Figure 3:**
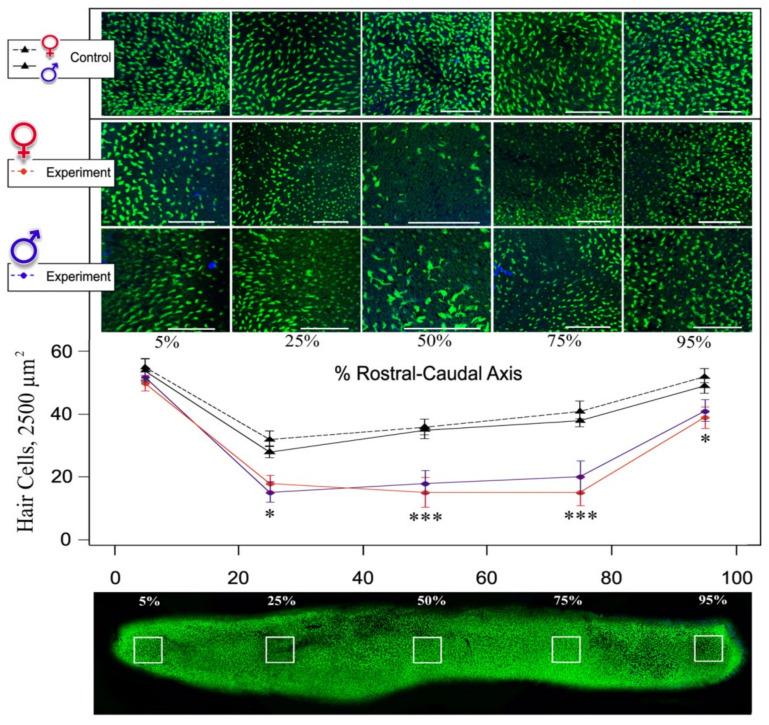
Effect of noise exposure on phalloidin-labeled auditory epithelium of stone sculpin males and females. The bottom image shows the five areas of hair cell counts along the rostral-caudal axis of the saccule from the most rostral (5%) to the most caudal (95%). Data are presented as mean ± standard error. Statistically significant differences are observed within 25–95% rostral-caudal areas between control and experimental samples, both male and female, and indicated with asterisks: * *p* < 0.05, *** *p* < 0.001 (2-Way ANOVA, Tukey HSD test).

Males and females of the control stone sculpins exhibited standard hair cells with intact stereocilia bundles and single kinocilia, previously observed in stone sculpins from the natural environment [101]. Noise-exposed fish had hair cells that were missing individual stereocilia or lacked stereocilia altogether, exposing the cuticular plates. We also found stereocilia fusion similar to previous descriptions of the hair cell damage caused by ototoxic antibiotics [102]. Because adequate discrimination of the listed types of cellular damage was complicated, we focused on quantifying structural damage based on the density of the saccular hair cells. The density of the saccular hair cell bundles differed significantly between control and noise-exposed stone sculpins, and we also observed a tendency for more hair cell loss in females, although there were no statistically significant differences between males and females in either controls or experiments (Figure 3).

In contrast to no damage in the control fish, at least minor or localized hair bundle loss was observed in all exposed specimens. Thus, the effect of hair bundle loss in five different areas of the inner ear (5%, 25%, 50%, 75%, and 95%) varied depending on the specific fish exposed to noise, but there were some common patterns (Figure 3). In both males and females, the highest hair cell loss occurred at the 25–95% rostral-caudal areas, where sparse hair cells and bundleless hair cells (cuticular plates without stereocilia) were observed (2-Way ANOVA, F_3,180_ = 85.45, *p* < 0.05, followed by Tukey HSD test). This can be explained by the fact that the saccule is tonotopically organized in teleost species, with frequency affecting the rostral and caudal of the saccule in a graded manner [71].

We hypothesize here that the cellular changes in both sexes of noise-exposed stone sculpins are the consequences of the oxidative stress described above, the past presence of which was evident at the blood level and led to more severe current consequences in females. Although the mechanisms of hair cell damage are complex, they involve the accumulation of reactive oxygen species [103], the main generator of which in cells are mitochondria, as mentioned above. Loss of mitochondrial membrane integrity results in the release of reactive oxygen species into the cytoplasm and may also lead to increased production of free radicals [104]. Production of free radical species has been previously observed in the inner ear of other species following the exposure to damaging noise levels [105,106] and after the treatment with ototoxic antibiotics [107]. Thus, the results of our analyses indicate that continuous noise affects hair cell loss in both male and female stone sculpins. The hair cell damage detected in this study likely resulted in hearing loss, as other studies had shown that hair cell damage led to a temporary threshold shift in fishes [91,108]. Furthermore, the previous study revealed the ultrastructural rearrangements in the olfactory receptor cells of Baikal sculpin males directed for the shutdown of pheromone perception during the paternal care period [109]. Therefore, we hypothesize that evoked sensory deprivation as an effect of hearing loss may also influence the occurrence of stress during the post-spawning period, as hearing and visual perception should be particularly activated in males during this time.

The loss of hair cells even in poor-hearing sculpins, especially males, could be due to a more rapid accumulation of noise-induced stress levels or the lack of prolonged recovery periods during continuous noise exposure. Actually, free radicals can be observed within hair cells long before any signs of damage are evident [110]. However, reactive oxygen species have been detected in the inner ear even seven to ten days after the noise exposure [111]. Such persistent oxidative stress likely induces progressive epithelial damage. Indeed, hearing loss in fish had been previously shown to be affected not only by noise intensity but also by the duration of noise-induced stress [5,112]. Continuous noise (e.g., in heavily trafficked areas) can have the greatest impact on stress and hearing loss, suggesting that it also has the strongest effect on fish reproduction [5] and even activates gene expression programs that can be strong determinants of senescence [103].

### 3.3. Noise Forces Accelerated Senescence Differently in Stone Sculpin Males and Females

The noise-induced molecular stress response in stone sculpins has been estimated using methods to measure telomerase activity and telomere length, which serve as markers of an organism’s physiological state [62,63]. It is noteworthy that telomere length and telomerase activity are differentially regulated and have divergent patterns of change during cell functionality; thus, a correlation between these traits is not obligatory [113,114].

**Figure 4:**
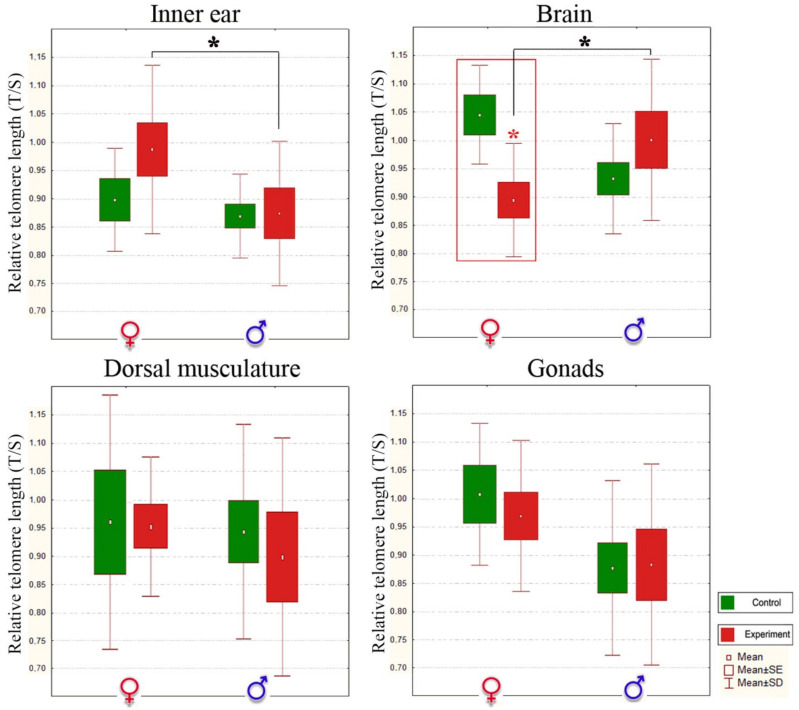
Relative telomere length (T/S) in control and noise-exposed stone sculpin males and females. T—concentration of telomere DNA, S—DNA concentration of reference gene, SD—standard deviation, SE—standard error. A statistically significant decrease in telomere length was observed in the brain of females between control and experimental samples and marked with a red asterisk. Statistically significant differences between the sexes in the experimental group are shown with a black asterisk: * *p* < 0.05, Kruskal-Wallis test.

**Figure 5:**
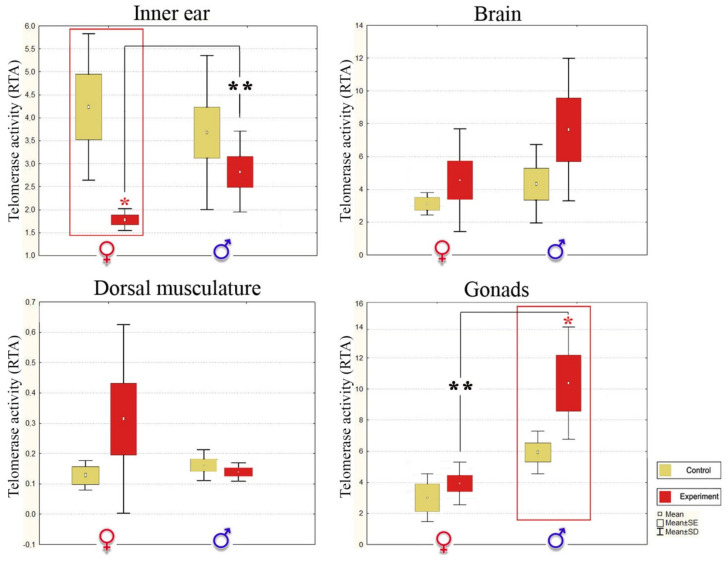
Relative telomerase activity (RTA) in different tissues of control and noise-exposed stone sculpin males and females. SD—standard deviation, SE—standard error. Statistically significant telomerase activity decrease was observed in the female auditory epithelium (inner ear), while a significant telomerase activity increase was recorded in male gonads upon noise exposure, marked with a red asterisk. Statistically significant differences between the sexes in the experimental group are shown with black asterisks: * *p* < 0.05, ** *p* < 0.01, Kruskal-Wallis test.

Interestingly, the telomerase activity was significantly lower in dorsal musculature than in other organs of both control sexes (Kruskal-Wallis test, *p* < 0.05). Moreover, there were no statistically significant changes in this tissue in noise-exposed fish (Table 3, Figure 4 and Figure 5), so we can consider dorsal musculature as a control non-target tissue for acoustic stress.

A statistically significant decrease in the telomerase activity of the inner ear tissue (auditory epithelium) and a shortening of telomere length in the brain (medulla oblongata) were observed after noise exposure only in females (Kruskal-Wallis test, *p* < 0.05, Figure 4 and Figure 5). The brain responds rapidly to environmental stresses, interacts intensively with neuroendocrine and immunological systems, and endures alterations in structure and function [115]. Overall, these data suggest that noise exposure leads to brain cell senescence in stone sculpin females.

Oxidative stress that promotes the erosion of telomeres is a likely mechanism by which mitochondria accelerate the senescence process [61]. In particular, we hypothesize that excessive noise exposure can “prime” nerve target tissues for damage where, over time, the effects of oxidative stress may overwhelm antioxidant defenses and cause long-lasting damage.

In stressed males, there was no significant deleterious difference in the telomerase activity and shortening of telomere length in nerve target tissues (Kruskal-Wallis test, *p* > 0.05, Figure 4 and Figure 5). This sex-dependent difference in load might be related to accelerated cellular aging in females and lower stress-related long-term risk in males, possibly through enhancing mitochondrial antioxidant capacity. Moreover, an increase in the telomerase activity was detected in male gonads (Kruskal-Wallis test, *p* < 0.05), indicating the importance of maintaining telomere length in male reproductive cells. These effects may be explained by the unique strategy of the stone sculpin males. Particularly, in the context of reproductive behavior at the stage of caring for offspring and maintaining their viability, stone sculpin males play a more important biological role, spending more energy and resources. Thus, we assume a stress resistance mechanism and lower stress-related long-term risk in males of this species. In other animal species, there are some scenarios in which an organism’s reproductive axis has evolved sensitivity or insensitivity to the suppressive effects of stress [23,116,117,118]. The testes of dominant males of the wild baboon, *Papio* spp., especially are less vulnerable to the disruptive effects of cortisol than those of subordinate males [108]. As in salmonids, glucocorticosteroid excess appears to play a role in programmed death [23]. Moreover, corticotropin-releasing-hormone-binding protein, an antagonist of the stress hormone, is specifically expressed in oxytocin receptor interneurons and produces an anxiolytics effect in male but not female mice, *Mus musculus* [118]. Therefore, we can infer the existence of stress suppressive mechanisms in stone sculpin males, although this requires further detailed studies.

## 4. Conclusions

In this paper, we consider Lake Baikal a natural laboratory where acoustic experiments can be simulated focusing on pure adequate control. Such HIREC modeling not only provides information about the potential consequences under anthropogenic pressure but also can help identify the natural mechanisms of stress resistance in different species, including those related to sex. We present here a molecular and cellular assessment of the sex-dependent effects of increased noise exposure in the stone sculpin that is capable of vocalization and lives in Lake Baikal and other noisier waters. This study reveals that anthropogenic noise at mean sound pressure levels of 160–179 dB re 1 μPa (SPL_pk-pk_) damages hair cells in the macula sacculi, causes changes in red blood cell cytometric parameters in both males and females, and also alters telomere length and telomerase activity in a sex-specific manner.

Thus, we observed increased oxidative stress, the long-term consequences of which are clearly visible in changes in blood morphology: through activation of functionally active mitochondria in males due to an increase in cellular energy cost and a decrease in energy cost due to resource depletion of an organism in females. Second, noise exposure caused morphological damage to macula sacculi hair cells and changes in hair bundle density in both males and females. We consider increased oxidative stress one of the most important possible mechanisms of nervous tissue damage, which triggers pathological reactions that damage the cell and lead to the initiation of apoptosis, a genetically programmed cell death, in macula sacculi. Finally, oxidative stress during the exhaustion phase of the organism could lead to changes at the molecular level that result in cellular aging (senescence), as observed, for example, in the shortening of telomeres in the stone sculpin females. The condition of females could apparently be affected by strong physiological changes during spawning. Thus, the results testify the stone sculpin females appeared to experience excessive molecular stress (distress) and were at the level of exhaustion based on the classical stress curve presented previously by Selye [22]. At the same time, the medulla oblongata neurons and auditory epithelium of stone sculpin males appear to be less sensitive to acoustic stress at the molecular level, indicating the existence of stress-buffering antioxidant factors. Thus, the stone sculpin males showed adaptive recalibrations that can be described here as a low allostatic load. These effects can be explained by the unique strategy of offspring maintenance in stone sculpin males and their biological role in reproductive behavior within the species. Finally, we conclude that the approach conducted can provide a reliable assessment of noise-induced stress in fish.

Additionally, we were not previously aware of any studies that addressed the effects of anthropogenic noise on the gonads of fish. However, it has been previously suggested that it is important to investigate potential noise effects in fish gonads [5]. In the current study, we investigated the issue of possible senescence of this tissue in noise-exposed male and female stone sculpins. We found no direct effect of acoustic stress on gonadal telomeres in both males and females, but we did detect an increase in the telomerase activity in male gonads. First, female oocytes are considered more conservative [23] than male gonads (in other words, gonadal telomere length in males must be actively maintained), so the increase in the male telomerase activity was most likely an adaptive effect of the overall stress state of males. Since telomerase is known for its ability to restore telomeres in emergencies, such an adaptive response might indicate the importance of maintaining telomere length in male reproductive cells. On the other hand, we need to clarify here that telomerase is a multifunctional enzyme and may be involved in stress without being directly involved in telomere maintenance [119]. Furthermore, we hypothesize that males might also switch from the “adaptive” to the “exhaustion” phase under prolonged stress, possibly leading to high mortality, unexpected effects, or senescence. We emphasize that future studies should investigate the possible “accumulative” and “delayed” effects of noise-induced stress in fish.

The obtained and future findings will help to elucidate the links between noise exposure, long-term sex-related changes in fishes, and the possible further evolutionary success of a species. Furthermore, we suggest the occurrence of a possible stress phenotype as a result of noise exposure within different species and populations in the natural environment, although this requires further confirmation. In addition, stress from noise may or may not interact with other stressors, e.g., rising temperatures may affect spawning habitats, making it critical to examine noise in a multi-stress context [1,5,8,120]. We hypothesize that clean control is of particular importance in this context.

The obtained results of these studies may help to understand and mitigate the effects of increased anthropogenic noise under different natural conditions and contribute to the development of effective environmental management practices.

## Figures and Tables

**Table 1:** Comparison of stone sculpin blood profiles (percentage of the total blood cells) in control and following seven days of noise exposure.

Blood Cells ^1^, %		Control		Noise-Exposed	
		♂	♀	♂	♀
**Peripheral blood**					
Erythroblasts ^2,^*		26.69 ± 3.86	33.38 ± 0.56	26.21 ± 2.9	23.32 ± 4.3 ^3,^**↓
**Mature red blood cells**		73.31 ± 3.9	66.62 ± 0.60	73.79 ± 2.89	76.68 ± 4.82 ^3,^**↑
**White blood cells**	Lymphocytes ^2,^**	92.11 ± 6.04	91.90 ± 5.3	94.10 ± 1.6	87.68 ± 7.7 ^3,^**↓
	Monocytes ^2,^***	0	0	0.41 ± 0.04 ^3,^***↑	0
	**Immature n** eutrophils ^2,^***	6.17 ± 0.35	2.72 ± 0.14	2.29 ± 0.13 ^3,^***↓	5.73 ± 0.3 ^3,^***↑
	Polymorphonuclear neutrophils ^2,^***	1.72 ± 0.08	5.38 ± 0.4	3.20 ± 0.15 ^3,^***↑	6.59 ± 0.5 ^3,^**↑
**Neutrophils to lymphocytes** ratio, N:L ^2,^*		0.08	0.08	0.06	0.14 ^3,^*↑
**Hematopoietic organs: Kidneys**					
Blasts ^2,^***		3.46 ± 0.14	12.7 ± 0.22	9.68 ± 0.36 ^3,^***↑	5.05 ± 0.25 ^3,^***↓
**Phagocytes**		58.00 ± 0.8	27.1 ± 0.24	30.1 ± 0.34 ^3,^***↓	29.1 ± 0.39
Lymphocytes ^2,^**		47.99 ± 8.94	56.25 ± 4.92	49.45 ± 5.19	58.48 ± 6.51
Plasmocytes ^2,^*		4.23 ± 0.24	3.95 ± 1.73	10.75 ± 0.32 ^3,^***↑	7.35 ± 3.59 ^3,^***↑
**Hematopoietic organs: Spleen**					
**Blasts**		6.63 ± 0.29	2.77 ± 0.15	5.53 ± 0.13	5.23 ± 0.33 ^3,^***↑
Phagocytes ^2,^***		43.8 ± 1.74	52.4 ± 1.44	47.6 ± 0.54 ^3,^*↑	36.4 ± 1.01 ^3,^***↓
Lymphocytes ^2,^**		47.07 ± 17.77	43.7 ± 13.10	41.85 ± 8.11	54.35 ± 8.36 ^3,^***↑
Plasmocytes ^2,^*		2.50 ± 0.12	1.13 ± 0.11	4.98 ± 0.15 ^3,^***↑	4.05 ± 0.23 ^3,^***↑

^1^ Phagocytes are presented by myelocytes, myeloblasts, monoblasts, and promonocytes. Lymphocytes include total lymphoblasts and lymphocytes. Data are presented as mean ± standard deviation. ^2^ Statistically significant differences between the sexes in the experimental group are shown immediately next to the name of the studied indicator with asterisks. ^3^ Statistically significant differences in the experimental groups compared with the control group are indicated with asterisks: * *p* < 0.05, ** *p* < 0.01, *** *p* < 0.001 (Kruskal-Wallis test). Arrows demonstrate a significant decrease (↓) or an increase (↑) in parameters compared to the controls.

**Table 2:** Cytometric parameters ^1^ of stone sculpin red blood cells in control and following seven days of noise exposure.

	Eccentricity ^2,^***	Nuclear Area ^2,^***	Cell Area ^2,^***	Cell Volume ^2,^***	Nuclear Volume ^2,^**	NCR ^2,^***
	Control					
♂	0.714 ± 0.003	23.74 ± 0.14	117.8 ± 0.68	1232 ± 11.6	112.1 ± 1.1	0.107 ± 0.001
♀	0.739 ± 0.004	23.51 ± 0.17	108.1 ± 1.24	1111 ± 18.8	110 ± 1.32	0.126 ± 0.003
	**Noise-exposed**					
♂	0.673 ± 0.003 ^3,^***↓	25.5 ± 0.13 ^3,^*↑	118.1 ± 0.75	1200 ± 11.3 ^3,^*↓	122.8 ± 1.04 ^3,^*↑	0.125 ± 0.002 ^3,^*↑
♀	0.704 ± 0.003 ^3,^***↓	24.7 ± 0.15 ^3,^*↑	122.4 ± 0.81 ^3,^***↑	1294 ± 13.4 ^3,^***↑	118.4 ± 1.19 ^3,^*↑	0.109 ± 0.002 ^3,^*↓
	EP ^2,^***	Hb, g/% ^2,^***	RBC ^2,^*	**BOC**	MCHC^2,^*	**K/A**
	**Control**					
♂	361.05 ± 65	7,92 ± 0.74	1.73 ± 0.02	12,52 ± 0,76	56,39 ± 8,7	1
♀	456.88 ± 44	6.41 ± 0.61	1.63 ± 0.03	10.36 ± 0.39	55.44 ± 0.39	1
	**Noise-exposed**					
♂	631.30 ± 72 ^3,^*↑	5.2 ± 0.38 ^3,^***↓	2.11 ± 0.06	12.31 ± 0.32	44.82 ± 5.88 ^3,^*↓	2 ^3,^*↑
♀	170.46 ± 47 ^3,^*↓	7.89 ± 0.30 ^3,^***↑	1.52 ± 0.04	12.49 ± 0.31 ^3,^***↑	53.01 ± 5.35	2 ^3,^*↑

^1^ NCR, nuclear-cytoplasmic ratio; EP (1 × 10^6^ μm^3^/L mm^3^), the enriched pool of functionally active mitochondria; Hb (g/L), hemoglobin concentration; RBC, the number of the red blood cells per 1 mL3; BOC (volume units), blood oxygen capacity; MCHC (μg/μL), hemoglobin content in red blood cells; K/A, hemoglobin cathode-anode fractions ratio. Data are presented as mean ± standard deviation. ^2^ Statistically significant differences between the sexes in the experimental group are shown immediately next to the name of the studied indicator with asterisks. ^3^ Statistically significant differences in the experimental groups compared with the control group are indicated with asterisks: * *p* < 0.05, ** *p* < 0.01, *** *p* < 0.001 (Kruskal-Wallis test). Arrows demonstrate a significant decrease (↓) or an increase (↑) in parameters compared to the controls.

**Table 3:** Relative telomere length (RTL) and telomerase activity (RTA) in different stone sculpin tissues ^1^.

**RTL ± SD**								
	Inner Ear Tissue ^2,^*		Brain ^2,^*		**Dorsal Musculature**		**Gonads**	
	**Control**	**Noise-Exposed**	**Control**	**Noise-Exposed**	**Control**	**Noise-Exposed**	**Control**	**Noise-Exposed**
♂	0.866 ± 0.07	0.871 ± 0.13	0.931 ± 0.09	1.001 ± 0.14	0.954 ± 0.21	0.792 ± 0.38	0.877 ± 0.16	0.881 ± 0.18
♀	0.898 ± 0.09	0.987 ± 0.15	1.045 ± 0.09	0.895 ± 0.10 ^3,^**↓	0.963 ± 0.23	0.956 ± 0.13	1.008 ± 0.13	0.968 ± 0.13
**RTA ± SD**								
	Inner ear Tissue ^2,^**		**Brain**		**Dorsal Musculature**		Gonads ^2,^**	
	**Control**	**Noise-Exposed**	**Control**	**Noise-Exposed**	**Control**	**Noise-Exposed**	**Control**	**Noise-Exposed**
♂	3.114 ± 1.34	2.782 ± 1.01	4.323 ± 2.39	7.630 ± 4.34	0.158 ± 0.05	0.133 ± 0.02	5.911 ± 1.37	10.408 ± 3.72 ^3,^*↑
♀	4.630 ± 1.60	2.113 ± 0.85 ^3,^**↓	3.130 ± 2.01	4.567 ± 3.82	0.127 ± 0.05	0.314 ± 0.25	3.074 ± 1.97	4.000 ± 3.98

^1^ Data are presented as mean ± standard deviation. ^2^ Statistically significant differences between the sexes in the experimental group are shown immediately next to the name of the studied indicator with asterisks. ^3^ Statistically significant differences in the experimental groups compared with the control group are indicated with an asterisk: * *p* < 0.05, ** *p* < 0.01 (Kruskal-Wallis test). Arrows demonstrate a significant decrease (↓) or an increase (↑) in parameters compared to the controls.

## Data Availability

The data that support the findings of this study are available on request from the corresponding authors.
